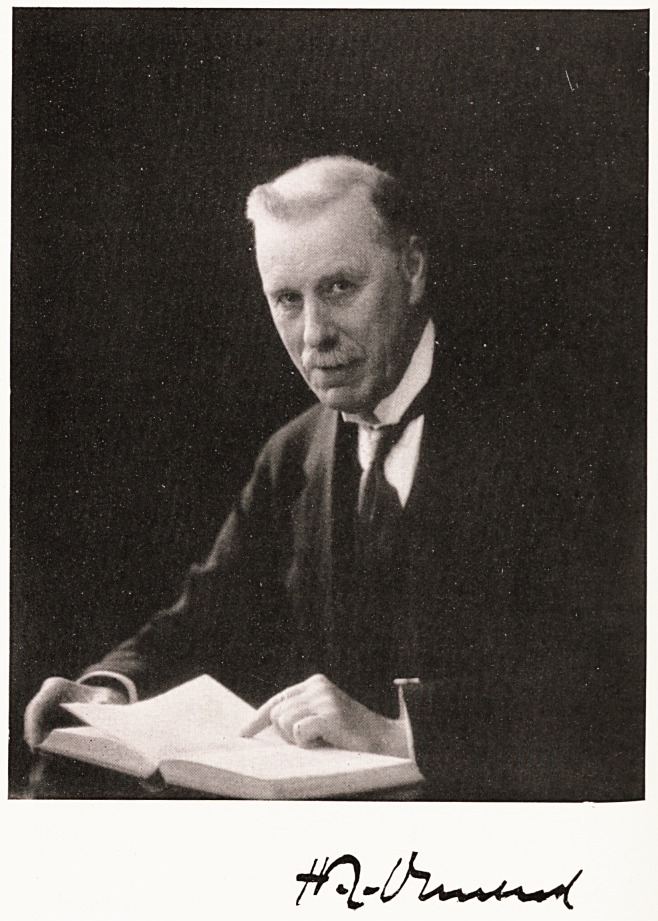# Henry Lawrence Ormerod

**Published:** 1932

**Authors:** 


### Obituary

### HENRY LAWRENCE ORMEROD, M.D., B.Ch., R.U.I.

We deeply regret to record the death of Dr. Henry Lawrence Ormerod, which occurred suddenly on 27th July, 1932. Dr. Ormerod was born in 1867 at Westbury-on-Trym, the eldest son of Henry Ormerod, surgeon for forty years at Westbury-on-Trym, and grandson of William Ormerod, surgeon, of Portland Square, Bristol. He was educated at Bristol Grammar School and won a scholarship at the Bristol Medical School and Bristol Royal Infirmary, where he gained the Suple Gold Medals for Surgery in 1888 and for Medicine in 1889. He obtained the diplomas of M.R.C.S. and L.R.C.P. London, in 1889, and soon after held the post of Junior House Surgeon and House Physician to the Bristol Royal Infirmary. In these offices he gave evidence of much ability and acumen, and his success in life was largely due to the thoroughness and assiduous attention with which he approached his early training. He started general practice at Westbury-on-Trym in 1895, and in order to qualify himself fully in this capacity he proceeded to the degrees of M.B., B.Ch., B.A.O. of the Royal University of Ireland in 1893 and M.D. in 1896.

In 1900 he married Marion Grace, eldest daughter of the late Canon Alford, of Bristol Cathedral.

Like his father before him, he possessed considerable powers of diagnosis, and was full of resource in the symptomatic treatment of disease which forms so important a part of the work of the family practitioner. Dr. Ormerod combined a sound professional knowledge with a gentle and equable disposition which gave composure to his patients and secured him many friends. The wisdom and fairness of his judgments gained for him the confidence of his colleagues, by whom he was regarded as an ideal family doctor.

His war service at Mr. R. E. Bush's hospital at Bishop's Knoll was an outstanding example of devoted effort unsparingly given, wholly unpaid and completely unrecognized by the War Office.

His medical appointments included :—

District Medical Officer and Public Vaccinator, Thornbury.
Medical Officer to the Post Office at Westbury-on-Trym.
Honorary Surgeon to the Bristol Eye Dispensary.
Certifying Factory Surgeon, and
Medical Officer at Brentry Colony.

Our Society showed its appreciation of his worth by making him President for 1928–29, and he was Chairman of the Bristol Division of the B.M.A. in 1927.

Ormerod was, in addition, one of the staunchest laymen in the Church of England. He was Chairman of the Cathedral Branch of the C.E.M.S., and Secretary of the Medical Service held annually in Bristol Cathedral. His personal views on the co-operation between the parson and the doctor may be illustrated by a resolution which he drafted not long before his death, for the Clerical and Medical Committee set up by the Bishop of Bristol: "Although from the strictly scientific point of view we have found no evidence of any cases of healing which cannot be paralleled by similar cures wrought by psycho-therapy without religion, and by instances of spontaneous healing which occur even in the gravest cases in ordinary medical practice, the Committee welcomes the co-operation between clergy and doctors, as by Religious Ministration the mental and moral outlook of the patient may be much improved and thereby the bodily power be greatly strengthened in its fight against disease."

He leaves a widow and two sons, to whom we extend our sympathy. His elder son, Dr. G. L. Ormerod, is continuing the practice at Westbury, thus maintaining the family tradition to the fourth generation.

## Figures and Tables

**Figure f1:**